# Bifunctional catalytic effect of Mo_2_C/oxide interface on multi-layer graphene growth

**DOI:** 10.1038/s41598-021-94694-4

**Published:** 2021-07-28

**Authors:** Seda Kizir, Wesley T. E. van den Beld, Bart Schurink, Robbert W. E. van de Kruijs, Jos P. H. Benschop, Fred Bijkerk

**Affiliations:** 1 Industrial Focus Group XUV Optics, MESA + Institute of Nanotechnology, University of Twente, 7522 NB Enschede, The Netherlands; 2 ASML Netherlands B.V., 5504 DR Veldhoven, The Netherlands

**Keywords:** Materials science, Nanoscale materials, Graphene, Synthesis of graphene

## Abstract

The role of the Mo_2_C/oxide interface on multi-layer graphene (MLG) nucleation during a chemical vapor deposition (CVD) process is investigated. During the CVD process, MLG growth is only observed in the presence of a Mo_2_C/SiO_2_ interface, indicating that the chemical reactions occurring at this interface trigger the nucleation of MLG. The chemical reaction pathway is explained in four steps as (1) creation of H radicals, (2) reduction of the oxide surface, (3) formation of C–C bonds at O–H sites, and (4) expansion of graphitic domains on the Mo_2_C catalyst. Different Mo_2_C/oxide interfaces are investigated, with varying affinity for reduction in a hydrogen environment. The results demonstrate a catalyst/oxide bifunctionality on MLG nucleation, comprising of CH_4_ dehydrogenation by Mo_2_C and initial C–C bond formation at the oxide interface.

## Introduction

Graphene is a two-dimensional material with unique properties such as high carrier mobility^1^, high strength^2^_,_ and high optical transparency^3^, which is advantageous for application areas such as transistors^4^, solar cells^5^, sensors^6^, and batteries^7^. Chemical vapor deposition (CVD) is currently the most promising method to synthesize large scale graphene for industrial applications^8,9^. Mo_2_C, from the transition metal carbide (TMC) family, is a leading candidate as a catalyst for uniform mono/multi-layer graphene synthesis due to its noble metal like catalytic activity, low cost, and high thermal stability^10–12^.

In our previous work, the growth of multi-layer graphene (MLG) was linked to the presence of pinholes in Mo_2_C thin films^13^. Such pinholes could expose the underlying oxide, which may play an important role in the graphene synthesis process. It is known from the field of catalysis that underlying oxide, commonly known as the “support layer”, can actively participate in catalytic reactions^14–16^. As an example, the influence of the support layer on carbon nanotube (CNT) synthesis is widely studied^17–19^, and it is found that the oxide interface causes initial CNT nucleation^18,20^. It is stated that ordered carbon formation on oxide is triggered by hydroxide and oxygen species^18,20^, which can be potentially important for MLG synthesis. Furthermore, Mo_2_C is a catalyst that shows different catalytic activity depending on the oxide under-layer (support) used^21,22^. Mo_2_C in combination with ZSM-5 zeolite supports causes aromatization of CH_4_, whereas this is not the case for only Mo_2_C or ZSM-5 separately^23^. This shows that both the catalyst and neighboring oxide can play a role in graphene synthesis, which to our knowledge has not yet been investigated.

In this study, we will demonstrate that the interface between pinhole-free Mo_2_C and SiO_2_ is triggering MLG nucleation upon the CVD process. We will introduce a model for explaining the MLG nucleation by catalyst/oxide bi-functionality. This model is based on the complementary role of MoCx (x = 0–0.5) for CH_4_ dehydrogenation and SiO_2_ for C–C bond formation after reduction with hydrogen. We will test this model by performing the CVD process in absence of hydrogen gas, as well as using different oxide (TiO_2_, Al_2_O_3_, MgO) interfaces, in which we expect a dependence of MLG nucleation on the oxide reducibility. The outcome of this study shows that the catalyst-oxide interface interaction is crucial for understanding the MLG synthesis on Mo_2_C thin films.

## Methods

In this section, the MLG synthesis process will be explained in three parts. In the first part, the thin film deposition process flow of Mo catalysts on Si/SiO_2_ substrates will be given. This process is known to result in pinhole-free Mo_2_C, which does not yield MLG growth upon subsequent CVD processing^13^. In the second part, the deposition/patterning of oxides will be explained in order to create an interface which is expected to play a role in MLG synthesis. In the final part, the applied CVD process for MLG growth, and characterization methods for analyzing synthesized MLG, will be described.

To remove possible surface contamination and the native oxide layer on top, *p*-type (100) single side polished silicon wafers are cleaned with ozone steam and HF, sequentially. To prevent Mo_2_C/Si interdiffusion, a 300 nm thermal SiO_2_ layer is grown on top of Si, by means of a dry oxidation process at 1100 °C. Directly before the sputtering process, the samples are cleaned with HNO_3_ to remove any organic contaminants on the sample surface. For the majority of samples, a Mo layer is deposited onto the SiO_2_ layer using magnetron sputtering, resulting in a pinhole-free Mo_2_C layer upon the CVD process described in ref 13. A nominal Mo layer thickness of 70 nm was used for all samples, because in separate experiments it was shown that thinner Mo layers (with pinholes) result in graphene layers with significant higher D/G ratios. This is explained by the strong reduction of the Mo crystallite size for thinner layers, which is expected to correlate to the formation of more pinholes and therefore significantly higher MLG nucleation density. In addition, ‘standard’ MLG thickness for 70 nm Mo layer (with pinholes) is around 11 nm corresponding to 32 layers, according to low energy ion scattering (LEIS) measurements (not shown here. After Mo deposition, a specific oxide top layer was deposited onto each sample using reactive sputtering. All sputter depositions are carried out in the same UHV deposition chamber, with a target to substrate distance of 300 mm and a base pressure of 10^–8^ mbar, using the conditions given in Table 1.

**Table 1:** Overview of the used sputtering parameters.

Material	Deposition pressure (E-4 mbar)	Ar (sscm)	O_2_ (sscm)	Current (A)	Voltage (V)	Target name (4 in.), supplier purity (%)
Mo	8.5	30	0	1	378	Mo, Robeko, 99.95
SiO_2_	7.8	20	20	0.25	306	Si, Sindhauser, 99.9999
TiO_2_	7.2	18	18	1	570	Ti, Robeko, 99.5
Al_2_O_3_	4.2	11	11	1.5	386	Al, Sindhauser, 99.99
MgO	7.9	20	20	1	172	Mg, Sindhauser, 99.95

**Figure 1:**
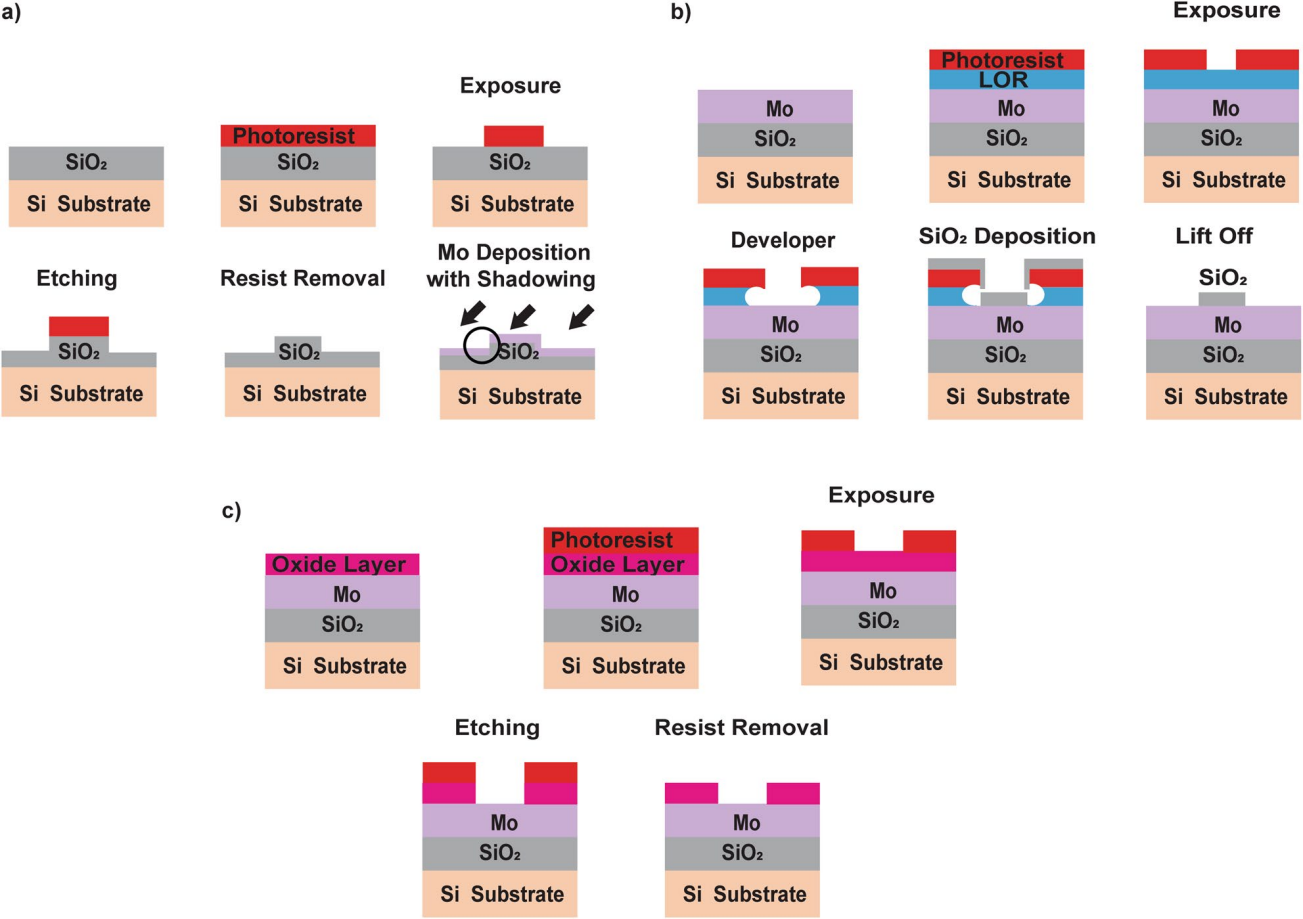
Schematic process of (**a**) SiO_2_ step fabrication via wet etching process and shadowed deposition of Mo, (**b**) lift off patterning of SiO_2_ lines on Mo layer, (**c**) wet etching patterning process for SiO_2_, TiO_2_, Al_2_O_3_ and MgO.

To test the impact of an exposed Mo/oxide interface on MLG growth, several procedures were developed based on optical UV lithography and including various wet etching/lift off steps, yielding a pattered oxide layer with lines and line spaces ranging from 1 to 10 micron, with length scales chosen similar to typical graphene domain sizes observed in previous studies. For creating an exposed Mo/SiO_2_ interface, two methods where developed. One method involves patterning the SiO_2_ layer before the deposition of Mo, as schematically shown in Fig. 1a. To fabricate a line step significantly higher than the thickness of the Mo layer, the thermal SiO_2_ layer is wet etched by buffered hydrofluoric acid (BHF) for 2 min resulting in a step of 150 nm. A Mo layer is then deposited at an off-normal angle, inducing a “shadow” in the deposited area on one side of the steps (shown by a black circle, Fig. 1a), leading to an exposed interface between Mo and SiO_2_. Another method to create an exposed Mo/SiO_2_ interface involves a lift-off process. Here, a ‘lift-off resist’ (LOR) and a top regular photoresist are deposited on top of the Mo/SiO_2_/Si layers. Subsequently, the LOR/photoresist is patterned upon UV exposure, a final 10 nm SiO_2_ layer is deposited, and the lift-off step is performed (as shown in Fig. 1b). In order to study and compare the influence of various other Mo/oxide interfaces on MLG nucleation and growth, a third structure was developed, where a 10 nm oxide layer (either SiO_2_, TiO_2_, Al_2_O_3_, or MgO) is deposited on top of Mo/SiO_2_/Si, as shown in Fig. 1c. To create an interface, the BHF wet etching process flow is used for oxide patterning, to avoid difficulties of removing lift-off resist for different oxides.

After fabrication of samples with exposed Mo/oxide interfaces, a CVD process is applied to all samples using a cold wall reactor system in presence of CH_4_, Ar and, H_2_ gasses at 1000 °C for 35 min, using the same parameters from our previous study^13^. To test the role of hydrogen in the MLG growth process, additional experiments were done where the same CVD process was applied, but without H_2_.

To characterize the structure of CVD deposited carbon, Raman measurements are performed, using a WITec alpha 300 system with a 0.9 NA objective and a 532 nm wavelength laser. The power of the laser is adjusted to 1 mW to avoid damage to graphene. The Raman spectra are recorded over a 10 by 10 micro meter area, and averaged for 9 spectra. I_d_/I_g_ ratios of graphene layers are obtained by processing of the Raman data with a MATLAB script using Lorentian peak fitting and normalization. The surface topography and elemental contrast imaging are carried out using a Zeiss MERLIN HR-SEM system with a voltage of 1.4 kV for improved surface sensitivity and in addition an EsB detector is used to make the nano-scale composition visible.

## Results and discussion

In our earlier work^13^, it is shown that the nucleation and growth (or absence thereof) of MLG on Mo_2_C was strongly related to the presence (or absence) of pinholes in Mo_2_C. From this observation, we hypothesize that the Mo_2_C/SiO_2_ interface can play an important, bifunctional, role by catalyzing different reactions for MLG growth. To test our hypothesis, in this section we investigate the role of the Mo_2_C/SiO_2_ interface in MLG growth on pinhole-free Mo_2_C.

### Effect of Mo_2_C/SiO_2_ interface on MLG nucleation

To create a system where within one sample both exposed and unexposed Mo_2_C/SiO_2_ interfaces are present, the structure as defined in Fig. 1a was prepared and subsequently exposed to the CVD process.

**Figure 2:**
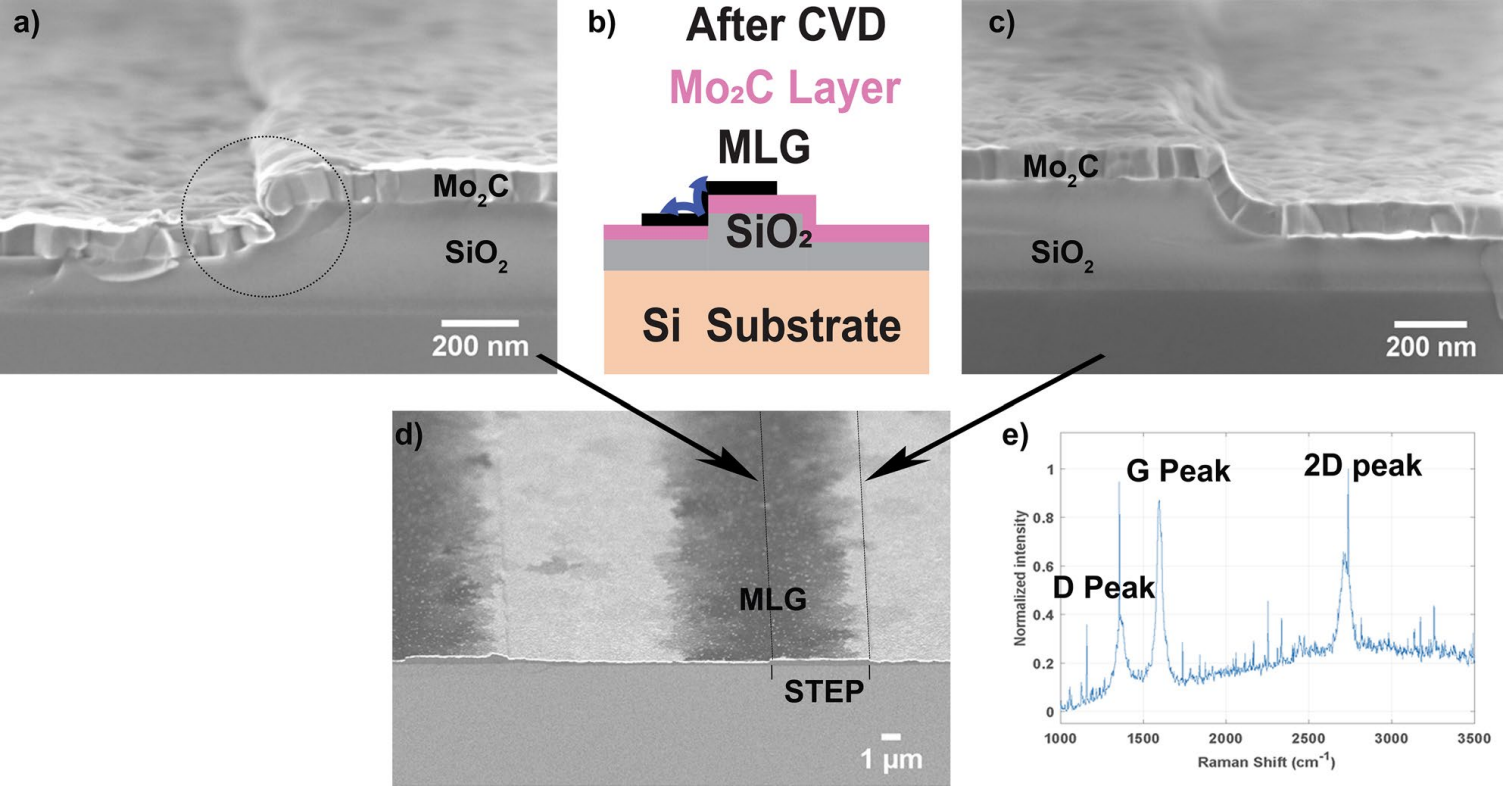
(**b**) Schematic image after CVD process is represented, where left side shows the MLG deposition from interface. SEM image after CVD process showing the cross-section from top view (**a**) Mo_2_C/SiO_2_ interface with dotted circle (left), (**c**) step fully covered with only Mo_2_C (Right) and (**d**) full view of the same step with elemental contrast mode imaging, in which MLG growth is shown around the left side of step with black contrast, and no graphene growth around right side of step with light contrast. (**e**) The Raman spectra after CVD process shows the deposition of MLG layers due to presence of D, G and 2D peaks.

In Fig. 2a, c the cross-section SEM images of the left and right side of the step after the CVD process is presented, showing (a) a Mo_2_C layer with an exposed Mo_2_C/SiO_2_ interface on the left side of the step, and (c) a continuous Mo_2_C layer on the right side of the step. Based on our hypothesis, MLG growth is expected where the Mo/SiO_2_ interface is exposed to the CVD process, as sketched in Fig. 2b. In Fig. 2d, the elemental contrast mode of SEM indeed confirms the presence of carbonaceous species (dark colour) centred on the exposed interface. The Raman spectrum is shown in Fig. 2e, confirming the characteristic peaks of MLG which are known as D, G, 2D peaks. This clearly suggest that MLG growth is initiated at the exposed Mo_2_C/SiO_2_ interface.

In the following section, we will propose a model explaining the MLG nucleation mechanism taking place exclusively at the exposed Mo_2_C/ SiO_2_ interface.

### MLG nucleation model

According to the d-band model, Mo has a strong interaction towards adsorbates such as CH_4_ leading to formation of a molybdenum carbide^24^, which is also known to occur upon the CVD process used for MLG synthesis^10,11^. In-situ formed Mo_2_C is generally considered as a catalyst for the MLG synthesis process, since it has noble metal like catalytic activity due to its modified band structure by compound formation^10,11^.

**Figure 3:**
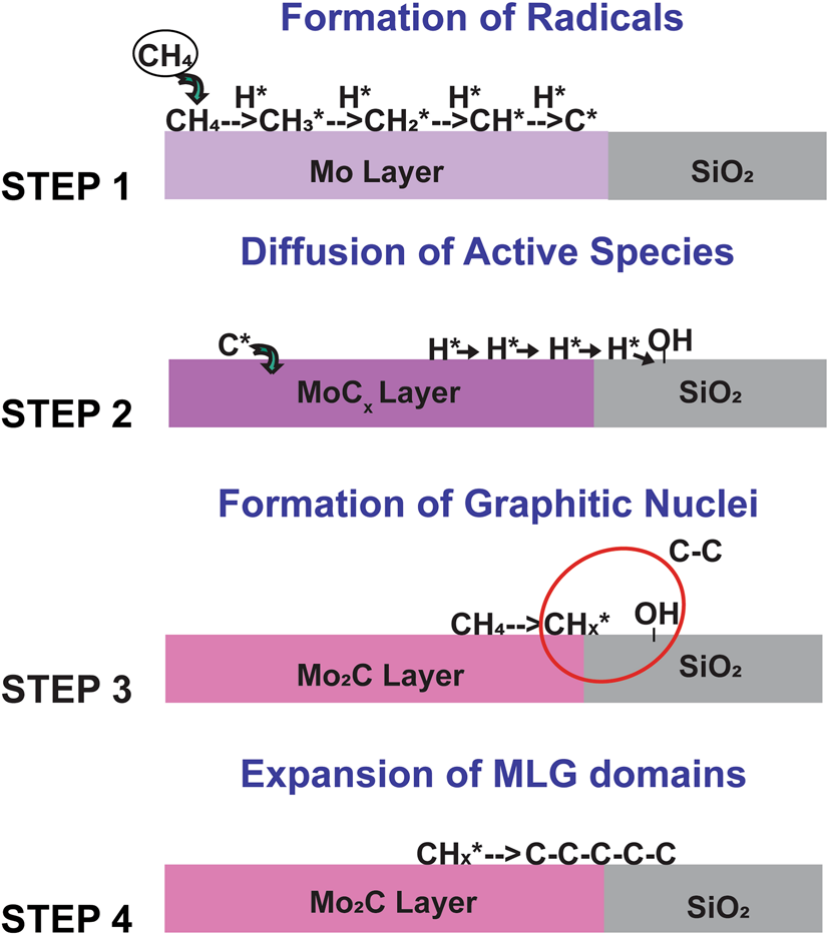
Schematic view of MLG nucleation model is explained in four steps.

In this section, a chemical reaction pathway will be proposed for MLG nucleation on Mo_2_C, with particular emphasis on the role of the exposed Mo_2_C/SiO_2_ interface, as indicated in Fig. 3.

#### Step 1: Formation of radicals

The first step of MLG nucleation is the adsorption and subsequent dehydrogenation of CH_4_ on the Mo surface. Mo has a high reactivity towards dehydrogenation of CH_4_ due to its d-band structure, resulting in formation of C* and H* radicals.

#### Step 2: Diffusion of radicals

The reaction pathway continues with the diffusion of C* and H* radicals, formed in the previous step. C* species diffuse into the Mo layer to form a stable carbide structure via Mo–C chemical bond formation^10,11^. Although some of the H radicals can diffuse into the Mo layer from grain boundary defects, this is expected to be minimal due to its low hydrogen permeability^25,26^. A part of the H* species is expected to diffuse over the Mo_2_C surface and onto the SiO_2_ surface, known as ‘Hydrogen spillover’^27^ (Fig. 3, Step 2). These diffused atomic H* species can cause a reduction of SiO_2_, especially at temperatures as high as 1000 °C^27,28^ causing activation of surface O–H sites.

#### Step 3: Formation of graphitic nuclei

Once the Mo_2_C catalyst is formed, the generation of CH_x_* radicals will continue on the catalyst surface via dehydrogenation of CH_4_^23^. These CH_x_* radicals can be adsorbed chemically on SiO_2_ surfaces at the interface through O–H sites, created in the previous step. This results in graphitic carbon formation via C–C coupling (Fig. 3, Step 3), which is a commonly known mechanism for graphene nucleation on SiO_2_ substrates^29–31^.

#### Step 4: Expansion of MLG domains

Once graphitic nuclei form at the interface between Mo_2_C and SiO_2_, MLG domains expand dominantly onto the Mo_2_C surface since the graphene growth rate on SiO_2_ is generally orders of magnitude slower^32,33^.

In summary, the MLG nucleation model proposed here is effectively driven by the Mo_2_C/SiO_2_ bi-functionality, where the oxide layer promotes the initial C–C bond formation whereas Mo_2_C causes CH_4_ dehydrogenation leading to the creation of radicals and expansion of MLG domains on its surface. Similar phenomena have also been reported for CNTs, where the oxide interface caused initial nucleation of CNTs and further growth continued on the catalyst by attachment of C species to the edge of the graphitic nuclei^18^. In the following section, the critical assumptions of the model based on support layer bi-functionality (steps 2 and 3) will be validated.

### Hydrogen spillover impact on MLG growth

In this section, we aim to study the importance of hydrogen spillover on the MLG growth process. In the MLG nucleation model in STEP 1, the expected source of the spillover H radicals is either the dehydrogenation of CH_4_ (CH_4_ ➔ CH_3_ ➔ CH_2_ ➔ CH ➔ C), formed upon the transition of Mo to Mo_2_C, or cracking of H_2_ (H_2_ ➔ H* + H*) during the CVD process. To investigate the role of H_2_ in the creation of OH sites (and thereby being responsible for the MLG nucleation), experiments were carried out with and without hydrogen gas.

**Figure 4:**
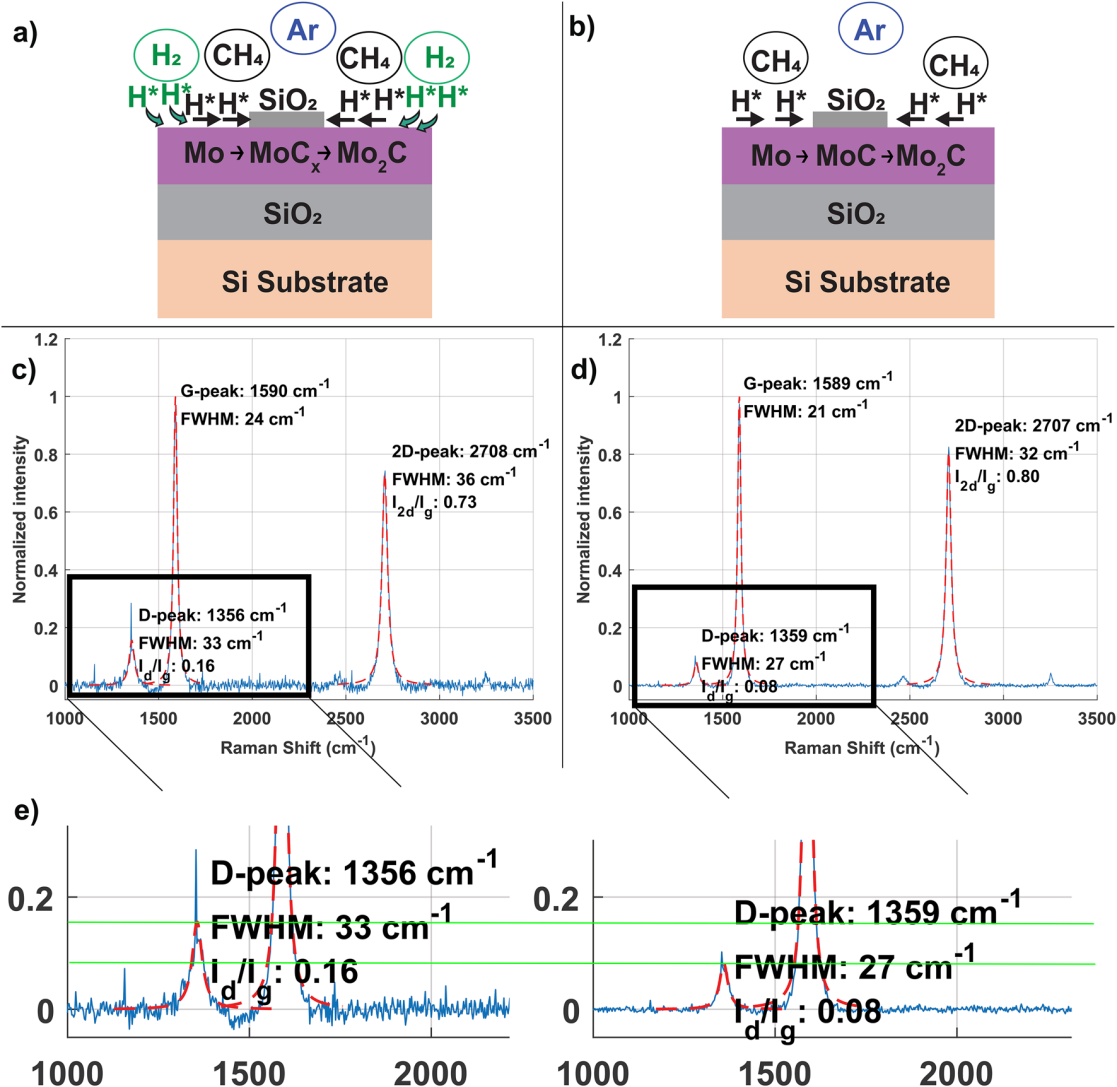
Schematic images showing the H spill-over during the CVD process for (**a**) standard CVD process, (**b**) CVD process in absence of external H_2_ gas. Corresponding Raman spectra depicting the MLG growth for both samples given as (**c**) sample with H_2_ gas in standard CVD process and (**d**) without, showing a difference in D peak. (**e**) Raman spectra zoomed in to D-peak region, comparing the I_d_/I_g_ ratios of the two samples, where for clarification a guide to eye was added (green line).

To fabricate a sharp Mo/SiO_2_ interface, two identical samples are prepared using lithography and lift-off processes, (See more details on the experimental section, Fig. 1b). The first sample is used as a reference in which our standard CVD process is applied (with Ar, H_2_, and CH_4_ gasses), whereas, for the second one, the CVD process is applied in absence of H_2_ gas, as shown schematically in Fig. 4a, b. The arrows indicate the transport (“spillover”) from Mo_2_C towards SiO_2_.

In Fig. 4c, d, the Raman spectra after the CVD process are shown. For both samples, D, G, and 2D peaks typical for MLG growth are observed^34^, suggesting that CH_4_ dehydrogenation upon carbide formation creates sufficient H radicals for MLG nucleation via spillover (MLG nucleation model, step 2). While the G and 2D peaks exhibit similar I_2d_/I_g_ ratios for both samples, the I_d_/I_g_ ratio is reduced significantly in absence of H_2_ gas in the CVD process (Fig. 4e). A lower I_d_/I_g_ ratio in the MLG growth process without H_2_ gas indicates the formation of larger domains, which suggests fewer nucleation sites^35^.

### Reduction of the support layer

An critical step in the proposed model is the creation of OH sites through reduction of the oxide layer by H*. To investigate the importance of the oxide reduction step, we compare samples with different oxides (SiO_2_, TiO_2_, Al_2_O_3_, MgO), chosen based on their difference in reducibility. Specifically, the order of reducibility in an atomic hydrogen environment is expected to be SiO_2_ > TiO_2_ > Al_2_O_3_ > MgO, with energies − 135,568 > − 120.763 > − 53.733 > − 15.632 kJ/mol O_2_^28^ with respect to their Gibbs free energy at 1000 K.

For interface formation, the oxide line patterns were created on top in order not to affect Mo_2_C crystallinity. A pattered surface with Mo/oxide interfaces was created for all samples using lithography and BHF wet etching (for more details, see experimental section Fig. 1c). Except for the sample with the MgO layer, all samples are patterned with a high yield. We believe that the sample with MgO continued to etch in the rinsing water after the BHF etching step, therefore only local patterns on sample could be analyzed.

**Figure 5:**
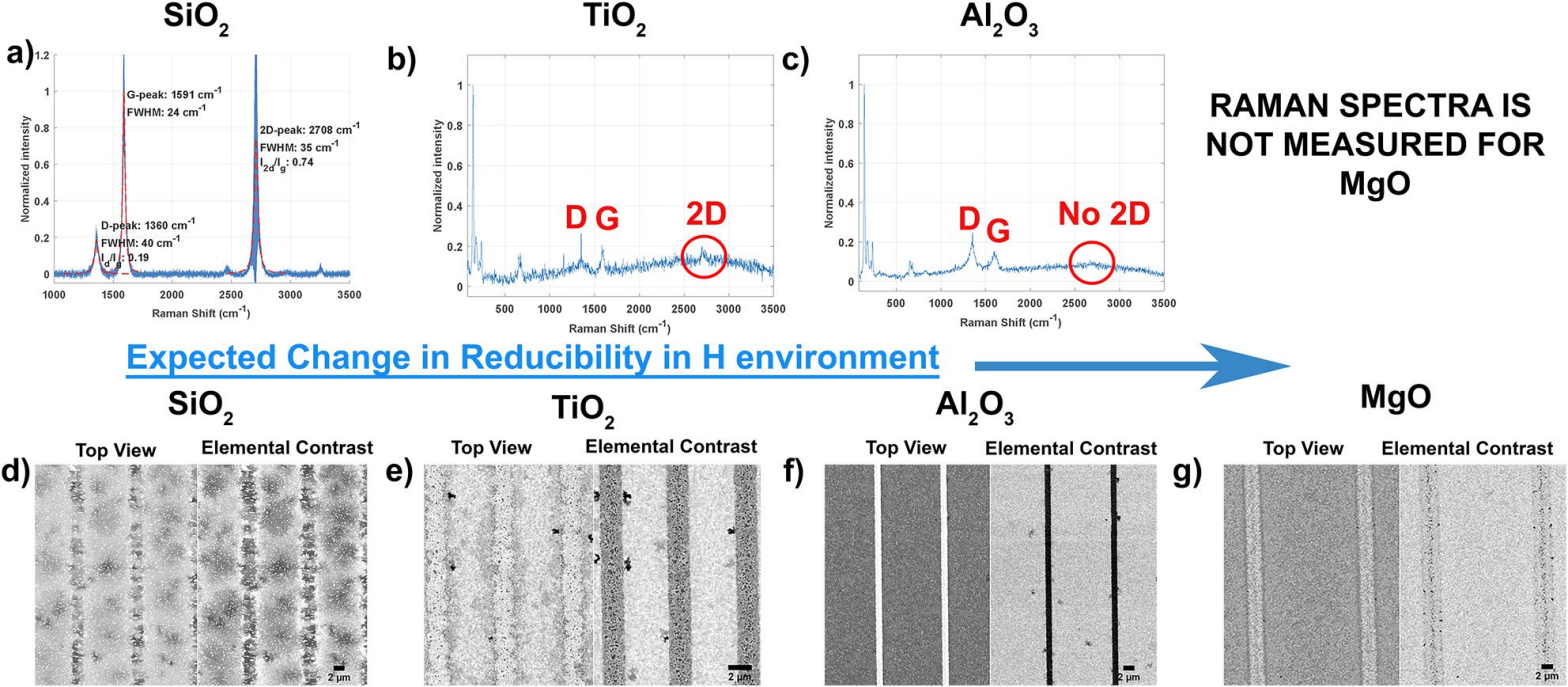
Reduction in the H environment follows the trend as SiO_2_ > TiO_2_ > Al_2_O_3_ > MgO^31^. (**d**) Schematic view shows the presence of O–H bonds with respect to reduction on oxide surface. Raman results are given, indicating MLG growth for SiO_2_ patterned sample (**a**), local growth with the appearance of small 2D peak for TiO_2_ patterned sample (**b**) and no growth for Al_2_O_3_ patterned sample (**c**). SEM results after CVD process is given with (**d**) SiO_2_, (**e**) TiO_2_, (**f**) Al_2_O_3_, (**g**) MgO lines on Mo_2_C, showing the C species with dark contrast in elemental contrast mode images.

In Fig. 5a–c, the Raman spectra after the CVD process are shown. Raman spectra indicate that sample with SiO_2_ lines showed strong MLG growth, whereas sample with TiO_2_ lines showed patchy growth and no growth is observed for the sample with Al_2_O_3_ lines. MLG growth is observed for SiO_2_ lines with the presence of D, G, and 2D peaks, showing similar spectra when compared to the lift-off patterned sample. The smaller appearance of the 2D peak for TiO_2_ suggests patchy deposition. No appearance of the 2D peak for Al_2_O_3_ indicates that there is no deposition of graphene. The local MgO patterns could not be traced back using Raman microscopy and thus no Raman spectra could be recorded. The Raman results clearly indicate that the reducibility of the oxide has a strong impact on graphene growth.

In order to semi-quantify the amount of carboneous species, the samples are analyzed simultaneously with the topography/elemental contrast mode of SEM, resulting in side-by-side images (Fig. 5d–g). Here, the elemental contrast mode shows the oxides and the carbonaceous species with darker contrast caused by their relatively low atomic number compared to the Mo-containing catalyst. The highest amount of carbon deposition is observed for SiO_2_ with dark contrast in between the lines. A smaller amount of carboneous deposition is observed on the edges of TiO_2_ lines. On Al_2_O_3_ and MgO samples, the carbon species are not observed. These SEM images correlate well with the observations from Raman, regarding the relation between oxide reducibility and graphene growth. These results therefore support our MLG nucleation model in which oxide reduction is a key step in creating OH sites required for triggering MLG nucleation.

The observed difference between SiO_2_ and TiO_2_ illustrated in Fig. 5a, b also suggests that the chemical reactivity for these oxides towards graphitization might be different, which is caused by a difference in the number of reactive sites and/or its strength^36,37^. In addition, the generation of intermediate volatile (reaction) products such as Si–O^38^, and, Si–H^39^, may lead to accumulation of graphitic carbon, similar to the graphene growth on SiC substrates^39–41^.

## Conclusions

In this report, the active role of the Mo_2_C/oxide interface on MLG nucleation is shown experimentally. A MLG nucleation model is proposed based on sequential chemical reactions, namely the formation of H species via CH_4_ dehydrogenation/H_2_ cracking, the migration of H species on the SiO_2_ surface (spillover), the reduction of the SiO_2_ surface, the activation of C–C bonds at O–H sites (as discussed in STEP 3- MLG nucleation model), and the expansion of graphitic domains onto Mo_2_C. It was shown that the dehydrogenation of CH_4_ is already sufficient to supply the H* species required for oxide reduction, whereas additional H* through cracking of H_2_ likely only increases the number of nucleation sites and reduces domain sizes. The Mo_2_C/oxide bi-functionality is further studied by using different oxide layers with varying reducibility. Increased reducibility leads to increased graphene growth, confirming the importance of the role of oxide reduction in the proposed model. The results shown here contribute to unraveling the role of the catalyst/support interactions that take place in graphene synthesis, as such the bifunctional catalytic effect of the oxide under layer. This could be also relevant for other catalyst/support combinations, yet more research is needed to deeper understand bifunctional chemical reactions at the atomistic level.
